# Medical Reform

**Published:** 1840-07

**Authors:** 


### MEDICAL REFORM.

THE consideration of the subject of Medical Reform becomes every day more interesting; and we hope we may add that the prospect of satisfactorily settling this great question becomes daily more cheering. We refer our readers to some important observations on this subject, as connected with medical education, to the conclusion of Art. VII., p. 200, et seq.; and we think we cannot do better, on the present occasion, than transfer to our pages from the Dublin Medical Press, one or two documents which are well deserving the attention of our readers. The first of these, the PLANS, was submitted by the Council of the Irish Association for the consideration of its members; and the REMARKS which follow are extracted from a letter addressed by C. T. CARTER, Esq., of Newcastle, to Dr. Maunsell, the active and intelligent secretary of the Association. With Mr. Carter's remarks we, in the main, accord; and especially in regard to the possibility and desirableness of moulding the existing corporations into harmonious union with the new structure which we trust is destined, ere long, to be raised for the common good of the profession.

### I. *Plans of General Medical Reform.*

1. The establishment, by law, of one faculty, having three branches, one in each of the capitals of the empire; such faculty to include all practitioners in medicine, both physicians and surgeons: each branch to be governed by a representative council, elected periodically by, and out of, the whole body of the faculty in each kingdom. The councils to have the power of making regulations for the government of the profession, and also of admitting members: no person being permitted to practise without being examined and licensed as a member of the faculty. The regulation of the three councils to be similar and uniform, general conferences being, from time to time, held in order to preserve uniformity. This "One Faculty" plan contemplates the establishment of a class of scientific apothecaries to be examined and licensed as such under the direction of the councils; also, that no practitioner "shall be permitted to sell drugs, or to compound medicines, unless prescribed by himself, or by others in consultation with him, and for his own patients, except in rural districts, and by special license." Mr. Donovan's proposal for establishing a college of pharmacy, might, with some modifications, be made to coincide with this portion of the "One Faculty" plan.

2. The effecting, in the first instance, of educational reform by the establishment by law of three boards, which alone should have the power of examining and licensing medical practitioners, thus superseding the bodies (eighteen in number) which at present grant degrees, diplomas, or licenses in the medical art. The appointment of such board should be in one of three ways: 1, by nomination by the crown; 2, by election by the profession at large; or 3, by selection by the crown from names returned by the profession. This plan is intended to have the effect of ensuring a sound and uniform minimum of education, without which no person should be permitted to practise; but it does contemplate any governing or protective institution.

3. A third plan of reform contemplates the continuance of the present corporations as examining bodies, but that they should be placed under the supervision of a board of control, empowered to superintend their operations and oblige them to preserve uniformity in their examinations and other modes of ascertaining the qualifications of persons seeking for their degrees or diplomas. That such board of control should not itself examine candidates, but should grant licenses to practise to those already examined by one or other of the existing corporations. That the license of the board should be obtained upon a mere production and verification of a certificate or certificates of qualification from one or more of the existing examining bodies; but that without such license from the board of control no person should be permitted to practise medicine within the British dominions. The appointment of the board of control might be made in one or other of the three ways pointed out as applicable to the appointment of the examining board contemplated in plan 2.

### II. *Remarks on the above Plans.*

I should very strongly recommend the Association to leave the third plan entirely out of the question. The second is unexceptionable as far as it goes, but if it contemplate educational reform alone it falls short of what is required in the present condition of medical affairs; but I conceive that the same tripartite medical council which, according to that plan, would regulate medical education, &c. might also act as a governing body for the whole profession. We want something more than uniformity of education, examination, and privilege. We require a governing body which shall understand and which shall represent the prevailing opinions and wishes of the profession, and which will on that ground be entitled to respect, not only from the profession, but in its dealings with the legislature. Under such auspices, the rights and privileges of men who had been licensed to practise would receive protection, public appointments wherein medical men are concerned would be properly bestowed, and matters relating to the public health, to medical jurisprudence and police, &c. would meet with adequate attention.

I am at a loss to imagine how uniformity of education and equal strictness of examination would be obtained by the third plan: I can see in it nothing but confusion. There is one insuperable objection to most of the existing bodies which at this time have power to confer degrees, diplomas, and licenses, in the same institutions which are concerned in educating having power to examine also, and to license their own pupils. I need scarcely dwell upon the impropriety of such an arrangement. . . . It is well known that gentlemen who have passed the ordeal of their own schools or universities have been subsequently rejected by other tribunals on the ground of incompetency. I will not detain you with a recital of the many temptations which, under existing circumstances, every college must have for granting the largest possible number of degrees, diplomas, or licenses. The examining and licensing body should, I humbly conceive, be wholly independent of any and every educational establishment, and the examiners should have stated salaries, their emoluments being in no way contingent on the number of candidates who might be either approved of or rejected. I think it would be next to if not quite impossible to ensure uniformity where so many bodies would be concerned in its promotion as are sanctioned by the third of the aforesaid plans, and am therefore of opinion that the examining and licensing of all candidates for medical practice should be committed to the three boards mentioned in plan number two. But how are these boards to be constituted so as to promote the foregoing objects, and at the same time to represent the interests of the medical profession, and conjunctively to act as a governing senate? In all long-standing institutions it is perhaps desirable to bring about any proposed change with as little convulsion as possible. Now there is no class of medical reformers, so far as I am aware, who are anxious to destroy existing corporations, or to deprive them in the slightest degree of any funds they may require for the prosecution of every useful and legitimate object. This should be clearly kept in mind, as it will tend to disarm, in some measure, that hostility which would naturally be excited by any attempt to procure a new system of medical government at the risk of annihilating existing institutions. But does it follow that in depriving these bodies of the power to examine and to license we should be adopting a course which would be ruinous to them? I have of late been endeavouring to show that uniformity in admission fees, as well as in other things, would raise an income far above what would be required for the support of the three boards, and a considerable portion of which might be handed over to the present corporations, independently of the augmented revenue which, under a liberal administration of their affairs, they might expect to receive from donations, bequests, subscriptions, &c.; and this, as was observed in the memorial of the Irish Association, "is the utmost that these bodies have a right to expect."

The establishment of the three boards (or I should rather say of the tripartite board, for the same regulations would prevail in each country), need not interfere with such duties as the present corporations are calculated to perform, and the efficient performance of which will, in any case, secure to them adequate pecuniary resources.

Respecting the construction of the three boards, three plans are proposed in the memorial just referred to, of which the third appears to me to be by far the most likely to meet with general concurrence, for the profession, I firmly believe, will never be satisfied until it be vested with power to control the administration of its own affairs. Witness the little interest felt in behalf of the University of London, wherein the senate is appointed wholly by the crown. At the same time it is perhaps desirable that any governing body which may hereafter come into existence should be placed in connexion with the secretary of state, although the members of it should not be entirely nominated by him. Existing institutions might perhaps, in the first instance, furnish materials in the formation of the national medical senatus or boards. The colleges might be opened to their members, all of whom, of a certain standing, being allowed to vote in the choice of officers, and the latter, after their election, being required to appoint delegates, who (subject to the revision of the crown), might form those central boards, to which it has been proposed to entrust the superintendence and direction of all matters relating to medical education, practice, and police, and to the preservation of the public health.... Some such scheme as the foregoing appears to be hinted at in Dr. Barlow's admirable paper on medical reform, in the January number of "Forbes's Review."

The acts of any one of the three boards would be tantamount to the acts of the whole, for their members would hold periodical conferences, and would be governed by reciprocal laws and regulations. The three boards would, in fact, during their session or congress, constitute the general medical council of the United Kingdom. To their deliberations might with safety be confided all matters of detail, amongst which I would include the "questio vexata" of "grades," for by such a representative and responsible council, distinctions and titles would scarcely be conferred, except upon just and proper principles; and provided they had such for their foundation, I, for one, should have no objection to "grades."....

In the expediency of making provision for a scientific and duly licensed class of apothecaries, I most cordially agree. The dispensing of medicine, through the medium of surgeons' apprentices, as practised in England, is, in my humble opinion, reprehensible in every point of view. I should like to see the medical practitioner, as far as it may be possible, freed from the harassing cares, and petty details, inseparable from trade, and devoting his exclusive energies to the higher aims and interests of his profession. Such a provision would put an end to the degrading custom, so generally prevalent on this side of the channel, of the medical attendant being remunerated by a profit upon the medicines sent to his patient—a custom which can never be sufficiently reprobated."

